# Salivary Diurnal Cortisol Predicts Post−Traumatic Stress Symptoms in Parents of Infants With Congenital Heart Disease

**DOI:** 10.1177/10998004231224791

**Published:** 2024-01-02

**Authors:** Amy Jo Lisanti, Fanghong Dong, Abigail Demianczyk, Maria G. Vogiatzi, Ryan Quinn, Jesse Chittams, Rebecca Hoffman, Barbara Medoff−Cooper

**Affiliations:** 1 School of Nursing, 6572 University of Pennsylvania, Philadelphia, PA, USA; 2 Research Institute, 6567 Children’s Hospital of Philadelphia, Philadelphia, PA, USA; 3 Center for Pediatric Behavioral Health, 22508 Cleveland Clinic Children’s, Cleveland, OH, USA; 4 Division of Endocrinology and Diabetes, 6567 Children’s Hospital of Philadelphia, Philadelphia, PA, USA; 5 Perelman School of Medicine, 6572 University of Pennsylvania, Philadelphia, PA, USA

**Keywords:** parent, stress, cortisol, trauma, mental health, critical care, congenital heart disease, intensive care

## Abstract

**Background:**

Parents of infants born with congenital heart disease (CHD) who require open heart surgery after birth are at risk for prolonged psychological distress. Even after their infants are discharged, parents may experience anxiety, depressive, and post−traumatic stress (PTS) symptoms; yet, it is unclear which parents are at greater risk for ongoing symptoms. The purpose of this study was to explore whether measures of the biomarker cortisol in parents during their infants’ postoperative period were associated with subsequent psychological distress symptoms at three−month post discharge.

**Methods:**

This was a prospective, longitudinal exploratory study of 40 parents of infants with CHD after open heart surgery using consecutive enrollment. Parents provided diurnal saliva samples for two consecutive days in the postoperative period. Six predictors were summarized and generated including waking cortisol, bedtime cortisol, cortisol awaking response, area under curve with respect to the ground (AUCg), cortisol index, and cortisol slope. Self−report outcome measures on anxiety, depressive, and PTS symptoms were collected three−months post−discharge. Linear mixed models examined the associations between each predictor and each outcome while accounting for within−dyad variance using an unstructured covariance matrix.

**Results:**

Cortisol AUCg was a predictor of PTS at three−months post−discharge (*β* = .34, *p* = .03, Cohen’s d = 2.05). No significant relationships were found with the other cortisol measures.

**Conclusions & Implications:**

Findings suggest that cortisol area under curve may help to identify parents at risk for increased PTS in the months following their infants’ hospitalization for cardiac surgery, serving as a foundation for future study in this area.

## Introduction

Parents of infants with congenital heart disease (CHD) often experience high levels of stress, particularly during their infants’ hospitalization for neonatal cardiac surgery and in the early months that follow (Golfenshtein et al., 2017; Lisanti et al., 2017; Torowicz et al., 2010). After discharge, parents often experience persistent symptoms of anxiety, depression, and post−traumatic stress (PTS; Woolf−King et al., 2017). It is important for healthcare providers to recognize and address the high levels of psychological distress symptoms experienced by parents of infants with CHD; however, few targeted and tailored interventions have been tested in this population (Kasparian et al., 2019). There remains a critical need for objective and precise measures that can identify which parents are at greater risk for ongoing symptoms and which interventions are most effective (Sood et al., 2021).

The hypothalamic−pituitary−adrenal (HPA) axis is a key physiological system that regulates the body’s response to stress (Sapolsky et al., 2000). HPA dysregulation has been linked to several mental health problems, including anxiety, depression, and post−traumatic stress disorder (PTSD; Ryan et al., 2016; Stickel et al., 2021; Wessa et al., 2006). Studies have shown that parents of infants with CHD have higher levels of stress compared to parents of healthy infants and parents of infants with other diseases (Golfenshtein et al., 2017; Lawoko & Soares, 2002). Parent stress can have a significant impact on HPA function, leading to HPA dysregulation. For example, Fecteau et al. (2017) found that parents of children with another stressful chronic illness, autism spectrum disorder, had higher levels of cortisol, a hormone regulated by the HPA axis, compared to healthy adults. The examination of parent cortisol as a biomarker of HPA function may help identify parents at risk for persistent psychological distress symptoms. Cortisol may also be used as an objective measure for testing interventions to reduce the negative impact of stress on parent mental health in CHD.

The Parent Stress and Resilience in CHD Model states that parent stress responses can be measured by physiologic biomarkers and psychological symptoms, including anxiety, depressive, and post−traumatic stress symptoms (Lisanti, 2018). Furthermore, the trajectories of parent stress can be influenced by experiences during the initial infant hospitalization and may exhibit sex differences across mothers and fathers (Lisanti, 2018). Using the Parent Stress and Resilience in CHD Model as a framework, this exploratory study aimed to determine whether diurnal measures of cortisol in parents of infants with CHD during the postoperative recovery from neonatal cardiac surgery are associated with subsequent psychological distress symptoms of anxiety, depression, and PTS at three−months post discharge.

## Methods

This was a prospective, longitudinal exploratory study that enrolled a convenience sample of 56 parents (28 biological mother–father dyads) of infants born with CHD who underwent neonatal cardiac surgery. Biological parent dyads were specifically enrolled to account for sex as a biological variable. Using consecutive enrollment methods, parents of postoperative infants were screened between August 2018 and October 2019 at one free−standing children’s hospital and approached at least 24 hr after their infant’s surgery while still hospitalized. Parents and infants were required to meet the following inclusion criteria: 1) parents at least 18 years of age; 2) parents able to read and speak English; 3) infants less than 30 days of age; 4) infants born at least 36 weeks gestational age and at least 2500 g weight. Parents and their infants were excluded if: 1) parents were taking any steroid preparations, sedatives, anti−anxiety, or other psychotropic drugs; 2) infants were diagnosed with other congenital syndromes or anomalies, listed for a cardiac transplant, or receiving end of life care. Due to the nature of this study as a pilot study, we aimed to recruit a homogeneous sample representing parents who experience the typical postoperative course in CHD and excluded parents of infants who would likely experience even greater stress due to their infants’ increased risk of morbidities and mortality from prematurity, other syndromes, heart failure requiring transplant, or palliative care. We acknowledge that non−English speaking parents or parents requiring medications for underlying mental health issues may also be at greater risk; however, this pilot study focused on the typical course for parents as a foundation for future research. This study received approval from the hospital’s institutional review board and parents provided their informed consent for themselves and their infants.

### Measures

**Figure 1.**
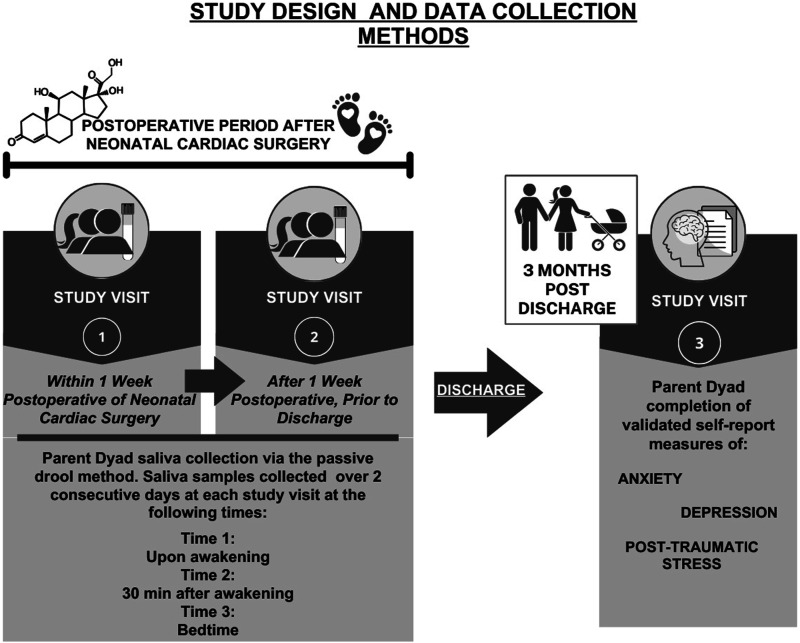
Study design and data collection methods.

The following measures were chosen based on the Parent Stress and Resilience in CHD Model and collected at one of three study visits (Figure 1).

#### Anxiety Symptoms

*The State Trait Anxiety Inventory* is a valid and reliable instrument that measures an individual’s tendency towards becoming anxious (Trait Anxiety subscale) and their number of anxiety symptoms (State Anxiety subscale; Spielberger et al., 1983) This study focused on the outcome variable of anxiety symptoms (State Anxiety) experienced 3−month post discharge in parents. The State−Anxiety subscale has been used in previous studies of parents of infants with CHD to measure the extent of anxiety symptoms experienced (Franck et al., 2005; Lisanti et al., 2017; Melnyk et al., 2004; Rychik et al., 2013; Turan et al., 2008). Scores range from a minimum of 20 to a maximum of 80, with higher scores indicating more anxiety symptoms.

#### Depressive Symptoms

The *Center for Epidemiological Studies−Depression (CES−D)* is a well−established instrument that measures the number of depressive symptoms experienced in the past week using a 4−point Likert scale (Radloff, 1977). The CES−D has been used to measure depressive symptoms in parents of infants with CHD (Lisanti et al., 2021a; Lisanti, et al., 2021b). A total score is calculated from the 20−item instrument resulting in a potential range of 0 to 60, with higher scores indicative of more depressive symptoms.

#### Posttraumatic Stress Symptoms

The *Impact of Events Scale – Revised (IES−R)* is another well−established instrument that assesses symptoms of PTS related to a specific traumatic event (Weiss & Marmar, 1997). The IES−R has been used with parents related to their infant’s critical illness (Cole et al., 2018). Total scores are calculated from the 22−item measure and can range from 0 to 88, with higher scores indicating more PTS symptoms.

#### Financial Strain

Financial strain was assessed using a 3−item index with 5−point rating scales on each of the following questions: 1. “How difficult is it for you to live on your total household income right now?” 2. “In the next two months, how much do you anticipate that you or your family will experience actual hardships such as inadequate housing, food, or medical attention?” 3. “In the next two months, how much do you anticipate having to reduce your standard of living to the bare necessities of life?” Cronbach’s alpha coefficients for this assessment has been reported to range from .86 to .88 (Vinokur et al., 1996).

#### Cortisol

Parents were provided with two study visits in the postoperative period while their infant was hospitalized to attempt saliva collection at wake up, 30−min after wake up, and bedtime for two consecutive days (Kraemer et al., 2006). For an hour prior to each sample collection, parents were asked to refrain from eating, drinking (except water), smoking, brushing teeth, or performing breastfeeding or mechanical breast pumping, as these activities may contaminate samples or influence cortisol secretion (Adam & Kumari, 2009; Gonzalez et al., 2009). Saliva samples were collected in SalivaBio Cryovials by Salimetrics® with Saliva Collection Aids using the passive drool method. Parents kept samples in a cooler with ice packs until picked up from the research team each day and then samples were frozen in a −20°C freezer until thawed for competitive cortisol immunoassay (Expanded Range High Sensitivity Salivary Cortisol Enzyme Immunoassay Kit, Salimetrics®). Samples were assayed in duplicate, according to the manufacturer’s directions, relative to a standard curve. Intra−assay coefficient of variability was 4.2%; and was 9.3% for high (1.0 μg/dL) and low (.1 μg/dL) controls. Of note, data collection for this study occurred over an entire calendar year and spanning all seasons. Therefore, we did not need to control for seasonal variation influencing cortisol (Miller et al., 2016).

### Data Analysis

Summary statistics were computed for participants’ demographic and clinical characteristics and presented as means and standard deviations for continuous measures, and as frequency and percentage for categorical measures. We dropped a total of eight samples with values greater than 3 standard deviations from the mean, considering them as outliers of cortisol and removing them prior to analysis as recommended by experts (Adam & Kumari, 2009; Stalder et al., 2016). Cortisol values were first aggregated by computing the mean across the two consecutive days for each timepoint (wakeup, 30 min, bedtime) within each study visit of sample collection attempts (Figure 1). For the analysis and summary of each cortisol measure, we used the first available evaluable data. If errors or missingness was found from a parent’s first attempt at collection at study visit 1, their second attempt at study visit 2 was evaluated for completeness and accuracy and used. Diurnal cortisol measures were computed for each participant. Cortisol Area Under the Curve (AUCg) with respect to the ground was computed using the trapezoid formula (Pruessner et al., 2003). Cortisol Awakening Response (CAR) was computed by taking the value of the 30−min post−awakening sample minus the awakening sample value (Adam & Kumari, 2009). Cortisol index was computed by dividing each CAR value by the respective awakening cortisol value (Angelhoff et al., 2019). Cortisol slope was computed by subtracting bedtime cortisol value from the wakeup cortisol value and dividing by the duration of time (hours) between wakeup and bedtime (Adam & Kumari, 2009).

Distributions of continuous outcome variables were assessed using histograms and normality (Q−Q) plots. We assessed the normality of the cortisol by conducting the Shapiro−Wilk test, and the results of this test indicated that the cortisol values were not normally distributed (*p* < .05). In response to the non−normality of our data, we applied a logarithmic transformation to the cortisol values to address the issue of skewness. This transformation was successful in achieving a more normal distribution of the data, as confirmed by a follow−up Shapiro−Wilk test (*p* > .05). The effect of each cortisol measure on each of the subsequent distress symptoms were assessed using linear mixed effects models which accounted for dyads nested among the same infant using an unstructured covariance matrix. Covariates on HPA axis functioning as identified in the literature included: age, education, BMI, race, financial strain, and tobacco use (Adam & Kumari, 2009; Champaneri et al., 2013). Additional covariates on psychological distress symptoms including timing of diagnosis (prenatal vs. postnatal) and length of stay were identified based on the Parent Stress and Resilience in CHD Model. Bivariate relationships between each outcome and covariates were assessed using simple regression. Covariates which exhibited significant bivariate association with an outcome variable were included as covariates in the respective multivariable model for each outcome. We estimated the effect size (Cohen’s d) according to Cohen’s definition (Weiner et al., 2017), to which the levels of small, medium and large effect size correspond to .2, .5 and .8, respectively. The significance level was set as alpha = .05 and all results are considered exploratory. Statistical analyses were conducted using R studio (version 2023.03.0).

## Results

Of the 56 participants enrolled in the study, 14 were lost to follow up, not providing data at the 3−month post−discharge time point, leaving a sample of 42 parents. Because this pilot study was exploratory in nature and focused on the typical course for parents as a foundation for future research, one additional dyad was withdrawn due to concerns that they experienced extreme and extenuating circumstances during their infant’s hospitalization that did not represent the typical course for parents. These concerns were confirmed when we identified that their values for all outcome variables (anxiety, depressive, and PTS symptoms) were extreme outliers and greater than 2.5 standard deviations from the mean (Miller, 1991). The decision to withdraw this dyad was consistent with our exclusion criteria to not enroll parents would likely experience even greater stress due to their infants’ increased medical risk or their own mental health issues. This resulted in our final sample of 40 parents.

None of the parents reported having Cushing’s disease, diabetes or other endocrine disorder that would influence cortisol secretion. Parents had a mean age of 33.4 (SD = 4.96), with 72.5% (*n* = 29) White, and a large proportion (*n* = 32, 80%) with some college education and above (Table 1). Mean BMI was 29.7 (SD = 6.25), with only 3 parents (7.5%), all fathers, reporting the use of tobacco. The mean wakeup cortisol value was .30 (SD = .17), and mean bedtime value was .086 (SD = .065). Mean scores of anxiety, depressive and PTS symptoms at three−month post−discharge were 39.7 (SD = 13.0), 10.9 (SD = 10.8), and 16.0 (SD = 14.0), respectively. No significant differences were found on all study variables between mothers and fathers.

**Table 1.** Descriptive Characteristics of the Study Participants.

	Mother (*N* = 21)	Father (*N* = 19)	p value	Overall (*N* = 40)
Age			.49	
Mean (SD)	32.9 (5.26)	34.0 (4.68)		33.4 (4.96)
Race			.61	
Non−white	7 (33.3%)	4 (21.1%)		11 (27.5%)
White	14 (66.7%)	15 (78.9%)		29 (72.5%)
Education			.24	
Highschool or lower	3 (14.3%)	4 (21.1%)		7 (17.5%)
Some college or college	9 (42.9%)	12 (63.2%)		21 (52.5%)
Graduate	8 (38.1%)	3 (15.8%)		11 (27.5%)
Missing	1 (4.8%)	0 (0%)		1 (2.5%)
BMI			.29	
Mean (SD)	28.6 (6.66)	30.8 (5.79)		29.7 (6.25)
Missing	4 (19.0%)	1 (5.3%)		5 (12.5%)
Finance strain			.54	
Mean (SD)	5.67 (2.52)	6.16 (2.46)		5.90 (2.47)
Tobacco use			.20	
No	21 (100%)	16 (84.2%)		37 (92.5%)
Yes	0 (0%)	3 (15.8%)		3 (7.5%)
Timing of diagnosis			1	
Postnatal	1 (4.8%)	1 (5.3%)		2 (5.0%)
Prenatal	20 (95.2%)	18 (94.7%)		38 (95.0%)
Wakeup cortisol			.48	
Mean (SD)	.321 (.163)	.283 (.172)		.303 (.166)
Bedtime cortisol			.45	
Mean (SD)	.0933 (.0616)	.0772 (.0694)		.0859 (.0649)
Missing	0 (0%)	1 (5.3%)		1 (2.5%)
Cortisol awaking response			.85	
Mean (SD)	.0743 (.186)	.0848 (.162)		.0793 (.173)
Cortisol area under curve			.72	
Mean (SD)	224 (108)	213 (84.3)		219 (96.6)
Missing	1 (4.8%)	2 (10.5%)		3 (7.5%)
Diurnal slope			.78	
Mean (SD)	.0153 (.0103)	.0142 (.0121)		.0148 (.0110)
Missing	1 (4.8%)	2 (10.5%)		3 (7.5%)
Cortisol index			.56	
Mean (SD)	.354 (.725)	.514 (.942)		.430 (.828)
Anxiety symptoms at 3 months post−discharge			.095	
Mean (SD)	42.9 (12.7)	36.1 (12.6)		39.7 (13.0)
Depressive symptoms at 3 months post−discharge			.30	
Mean (SD)	12.6 (11.4)	9.00 (10.1)		10.9 (10.8)
Missing	0 (0%)	1 (5.3%)		1 (2.5%)
PTS symptoms at 3 months post−discharge			.18	
Mean (SD)	18.8 (14.4)	12.8 (13.2)		16.0 (14.0)

Table 2 presents the association between cortisol measures in parents of infants during the postoperative period and subsequent psychological distress symptoms of anxiety, depression, and PTS at three−months post discharge. Unadjusted model results are presented in Supplemental Table 1. Cortisol AUCg was a predictor of PTS symptoms three months after discharge (*β* = .34, *p* = .03, Cohen’s d = 2.05), after adjustment. The complete model with all covariates is presented in Supplemental Table 2. The associations between cortisol CAR and cortisol index with PTS were significant in the unadjusted model but did not achieve significance in the adjusted model, although relationships demonstrated very large effect sizes in both models. There were also large effect sizes found between cortisol AUCg and anxiety (*β* = .35, *p* = .085, Cohen’s d = 1.51) that had a significant association in unadjusted model but did not reach significance in the adjusted model. Large effect sizes were also found between cortisol AUCg and depression (*β* = .42, *p* = .084, Cohen’s d = 1.69). Sex as a biological variable was not significant in any of the models.

**Table 2.** Linear Mixed Models of Anxiety, Depressive, and PTS Symptoms at 3−Month Post Discharge Regressed on HPA Axis Function in Parents During the Postoperative Period.

	Anxiety symptoms as an outcome^a^						Depressive symptoms as an outcome^b^						PTS symptoms as an outcome^c^					
	β	B	SE	CI	*p*	Cohen’s d	β	B	SE	CI	*p*	Cohen’s d	β	B	SE	CI	*p*	*Cohen’s d*
Wake up	.15	9.89	10.52	[−11.97, 31.75]	.374	.66	.06	2.67	9.15	[−15.96, 21.30]	.779	.22	.13	9.35	9.43	[−9.87, 28.56]	.351	.70
Bedtime	−.03	−4.9	29.1	[−66.68, 56.89]	.871	−.13	.39	54.3	36.08	[−21.30, 129.90]	.183	1.23	−.06	−10.09	24.36	[−60.76, 40.59]	.691	−.31
Cortisol awaking response	.29	18.34	10.4	[−3.28, 39.96]	.116	1.25	.18	7.58	8.5	[−9.73, 24.90]	.402	.67	.22	15.02	10.43	[−6.24, 36.28]	.188	1.02
Cortisol area under curve	.35	.04	.02	[−.0021, .075]	.085	1.51	.42	.03	.02	[−.00026, .064]	.084	1.69	.34	.04	.02	[.010, 0.073]	**.030**	2.05
Diurnal slope	.13	128.61	191.12	[−275.61, 532.83]	.523	.51	−.04	−34.84	154.46	[−356.46, 286.79]	.829	−.18	.22	250.69	165.49	[−91.95, 593.33]	.174	1.14
Cortisol index	.15	1.98	2.06	[−2.31, 6.27]	.365	.68	.09	.81	1.71	[−2.66, 4.29]	.648	.36	.19	2.74	1.82	[−.96, 6.45]	.169	1.07

*Note*.

^a^Adjusted for education and BMI.

^b^Adjusted for education, BMI, race, and length of stay.

^c^Adjusted for education, BMI, and Prenatal diagnosis.

## Discussion

This pilot exploratory study provides the first longitudinal examination of parent HPA axis functioning on parent psychological distress symptoms three−months after discharge from infant cardiac surgery. We found preliminary evidence that cortisol AUCg may be an important biomarker for predicting later distress symptoms. Cortisol AUCg represents the total daily output of cortisol secreted by an individual. Increased daily cortisol secretion in response to stressful events may place individuals at greater risk for prolonged psychological distress symptoms. Large effect sizes in cortisol AUCg were demonstrated for all outcomes, with a significant relationship found with PTS symptoms. Anxiety and depressive symptoms approached significance and may not have reached significance due to the limited sample size. We also saw medium to large effect sizes with CAR and anxiety, depressive, and PTSD symptoms. Findings can be used to calculate power analyses for future longitudinal studies examining psychological distress symptoms over time. Additionally, future interventional studies targeting parent psychological distress in this at−risk population may benefit from including salivary cortisol as an objective outcome measure.

Greater attention is being given in the critical care field to post−intensive care syndrome in pediatrics as well as in families of critically ill adults. A recent study of family members with a critically ill loved one in an adult ICU found that CAR predicted anxiety symptoms experienced by family members three−months after discharge (Beesley et al., 2018a). They found no association with diurnal cortisol and PTS and depression (Beesley et al., 2018b). Their study was limited by selection bias and by only capturing saliva samples on one day, therefore missing the day−to−day variability known to occur with diurnal cortisol. They also did not control for BMI, tobacco/alcohol use, or other factors potentially influencing HPA axis functioning, as we controlled for in our study. Additional research is needed to understand the physiologic stress on parents and family members during a loved one’s critical care hospitalization and the development or persistence of subsequent psychological distress symptoms, a morbidity associated with post−intensive care syndrome.

From a methods perspective, our study is strengthened by its longitudinal nature and the assessment of diurnal cortisol. Most studies examined relationships between HPA axis functioning and psychological distress symptoms at a single time point. For example, studies measuring diurnal cortisol and PTSD examined these relationships at one−time point after the trauma had already occurred and the individual was experiencing PTSD symptoms. Studies using this design have demonstrated discrepancies, with associations for PTSD occurring with lower cortisol levels, higher cortisol levels, and some with no relationships (Speer et al., 2019). It may be that the development of PTSD is related to the diurnal cortisol pattern at the time of the traumatic event and not at the time of later symptom development. More research is needed that measures the physiologic stress response during the traumatic event as opposed to examining later associations. This study addressed the demanding nature of cortisol assessment, demonstrating the feasibility of obtaining multiple samples throughout the day for two consecutive days in a highly stressed cohort of parents during their traumatic experience of having an infant in the ICU to examine HPA axis functioning and psychological distress symptoms. Our study was also strengthened by the inclusion of sex as a biological variable, including mother−father dyads in all models and accounting for the anticipated correlation of measures of parent dyads of the same infant.

Our pilot study has inherent limitations affecting generalizability and the findings should be considered exploratory. To minimize the burden on subjects, we did not control exact saliva sampling times or use objective methods to obtain sampling times. We did not require strict awakening and bedtime. This was due to the expected sleep disturbance experienced by parents of newly born infants as well as parents with a child in the ICU. Future studies should consider obtaining objective sleep data, as well as objective data capture of sample collection times. Future studies should also include larger, more diverse sample sizes, as findings cannot be generalized to single parents, non−English speaking populations, or gender minority families, all of whom have growing needs and may experience health inequities. We excluded families not representing the typical course for parents, such as those experiencing end of life or infant palliative care, infant heart transplant, or extreme social or extenuating life circumstances outside of the infant hospitalization experience. Future studies with larger sample sizes should include these diverse experiences to improve generalizability. Finally, we did not measure additional factors or events occurring between hospital discharge and three−month follow−up that could have influenced outcomes. Subsequent anxiety, depression, or PTSD could have occurred from other life events, social support, or the infant’s health care utilization post−discharge. Future studies should examine and control for these factors that may influence parents’ distress symptoms.

Despite these limitations, our preliminary findings suggest that diurnal cortisol may be a promising biomarker to incorporate in future research examining parent mental health in this vulnerable congenital heart disease population. Diurnal cortisol profiles seem to indicate long−term effects of PTSD symptoms and could be considered as a biomarker in future psychological interventions for PTSD symptoms in this population. Larger, multisite studies with more diverse samples can be powered on our findings and offer more definitive information regarding how diurnal cortisol may identify at−risk parents for subsequent psychological distress symptoms post−neonatal cardiac surgery.

## Supplemental Material

Supplemental Material - Salivary Diurnal Cortisol Predicts Post−Traumatic Stress Symptoms in Parents of Infants With Congenital Heart Disease Supplemental Material for Salivary Diurnal Cortisol Predicts Post−Traumatic Stress Symptoms in Parents of Infants With Congenital Heart Disease by Amy Jo Lisanti, PhD, RN, CCNS, Fanghong Dong, PhD, MA, Abigail Demianczyk, PhD, Maria G. Vogiatzi, MD, Ryan Quinn, MPH, Jesse Chittams, MS, Rebecca Hoffman, PhD, and Barbara Medoff−Cooper, PhD, RN, FAAN in Biological Research For Nursing.
