# Supplementary material for: Salivary Diurnal Cortisol Predicts Post−Traumatic Stress Symptoms in Parents of Infants With Congenital Heart Disease
Source: Biol Res Nurs. 2024 Jan 2;26(3):341–9. doi: 10.1177/10998004231224791 (PMC11131345; doi:10.1177/10998004231224791)
Supplement: Supplemental Material - Salivary Diurnal Cortisol Predicts Post−Traumatic Stress Symptoms in Parents of Infants With Congenital Heart Disease [file sj-pdf-1-brn-10.1177_10998004231224791.pdf]

Supplemental Table 1. Linear mixed models of subsequent psychological distress symptoms of anxiety, depressive and PTS symptoms at three-month post discharge regressed on measures of HPA axis function in parents of infants during the postoperative period. (Unadjusted model)

	Anxiety Symptoms as an outcome[a]					Depressive Symptoms as an outcome[b]					PTS Symptoms as an outcome[c]				
	β	*B*	*SE*	*p*	*Cohen's d*	*β*	*B*	*SE*	*p*	*Cohen's d*	*β*	*B*	*SE*	*p*	*Cohen's d*
**Wake up**	0.16	9.87	10.37	0.356	0.48	0.06	2.71	6.59	0.687	0.21	0.03	1.84	10.11	0.858	0.091
**Bedtime**	-0.08	-12.15	23.65	0.615	-0.27	0.22	30.78	24.06	0.222	0.68	-0.06	-10.49	21.24	0.629	-0.26
**Cortisol awaking response**	0.21	12.75	9.26	0.188	0.69	0.12	4.90	5.84	0.415	0.43	0.34	21.64	8.22	**0.018**	1.31
**Cortisol area under curve**	0.36	0.04	0.02	**0.038**	1.22	0.29	0.02	0.01	0.099	0.98	0.34	0.04	0.02	**0.048**	1.16
**Diurnal slope**	0.12	106.23	143.38	0.471	0.39	-0.01	-9.43	108.34	0.932	-0.048	0.06	57.29	140.82	0.690	0.21
**Cortisol index**	0.16	1.98	1.92	0.317	0.51	0.09	0.79	1.13	0.496	0.36	0.31	4.11	1.55	**0.017**	1.33

Supplemental Table 2: Linear mixed models of subsequent mental health symptoms of anxiety, depressive and PTS symptoms at three-month post discharge regressed on measures of HPA axis function in parents of infants during the postoperative period.

Predictors	*β*	B	SE	CI	p
(Intercept)	-0.43	34.99	13.85	[9.28, 60.69]	**0.022**
**Cortisol area under curve**	0.34	0.04	0.02	[0.010, 0.073]	**0.030**
Education_Some college or college	0.42	5.13	3.68	[-0.77, 16.26]	0.206
Education_Graduate	0.63	7.75	4.11	[-2.49, 12.75]	0.102
BMI	-0.10	-0.21	0.27	[-0.78, 0.35]	0.461

Congenital diagnosis	-0.47	-22.62	5.22	[-32.30, -12.94]	**0.001**
Mother-father dyad	-0.22	-5.429	3.97	[-13.640, 2.782]	0.213
